# Supplementary material for: Characterizing the Short- and Long-Term Temporal Dynamics of Antibody Responses to Influenza Vaccination
Source: medRxiv. 2025 Feb 27:2025.02.26.25322965. Preprint. [Version 1] doi: 10.1101/2025.02.26.25322965 (PMC11888507; doi:10.1101/2025.02.26.25322965)
Supplement: 1 [file NIHPP2025.02.26.25322965V1-supplement-1.pdf]

medRxiv preprint doi: https://doi.org/10.1101/2025.02.26.25322965; this version posted February 27, 2025. The copyright holder for this preprint (which was not certified by peer review) is the author/funder, who has granted medRxiv a license to display the preprint in perpetuity.
It is made available under a CC-BY 4.0 International license .

Supplemental Materials

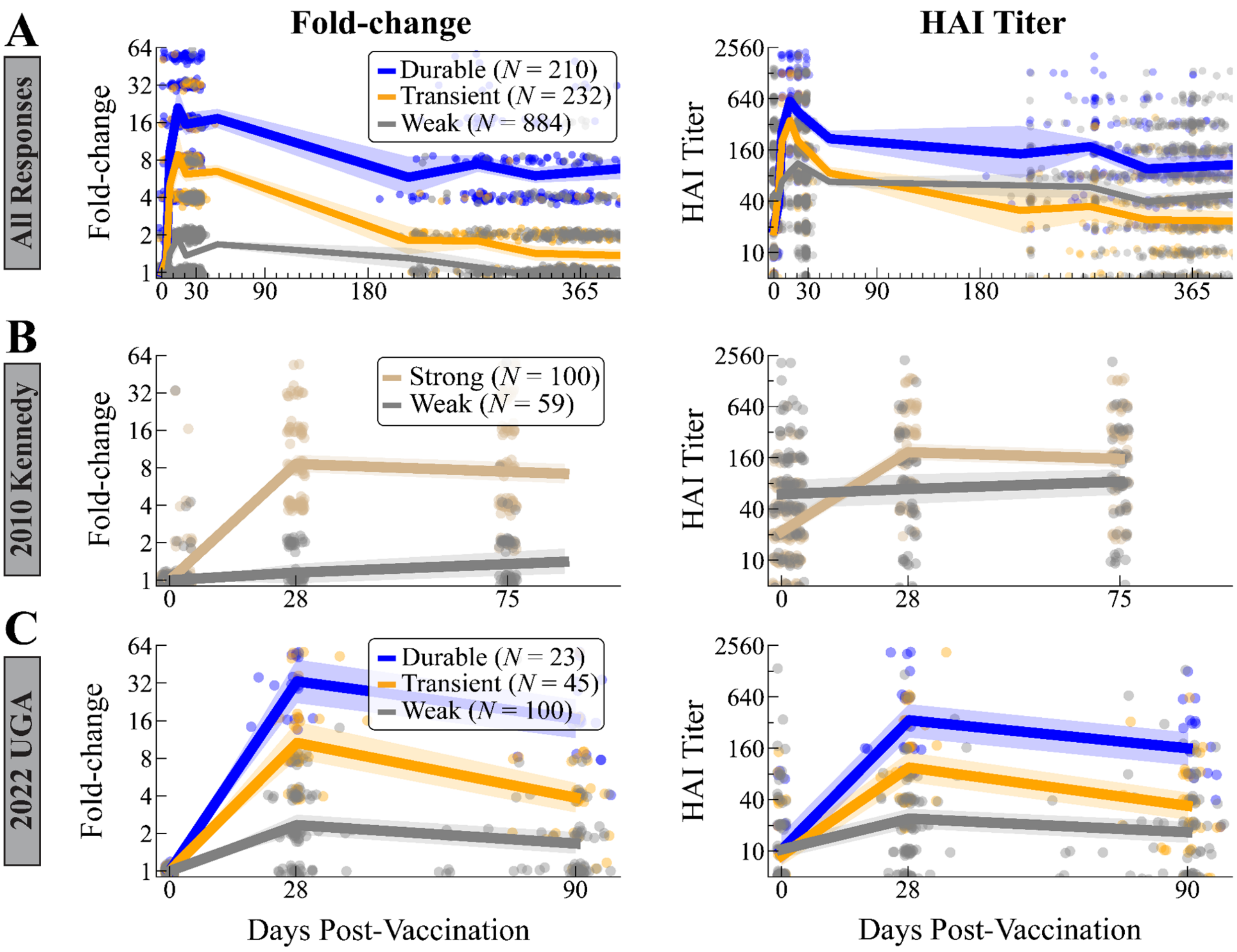

Figure S1. Comparing fold-change and absolute HAI titer. Fold-change [*left*] and absolute HAI titer [*right*] for durable, transient, and weak responders between (A) days 0-400 for all sera in **Table 1** (white background), (B) days 0-100 for 2010 Kennedy, and (C) days 0-100 for 2022 UGA. All plots are shown without the smoothing effects used in the main text. Legends apply to the left and right panels within each row.

medRxiv preprint doi: https://doi.org/10.1101/2025.02.26.25322965; this version posted February 27, 2025. The copyright holder for this preprint (which was not certified by peer review) is the author/funder, who has granted medRxiv a license to display the preprint in perpetuity.
It is made available under a CC-BY 4.0 International license .

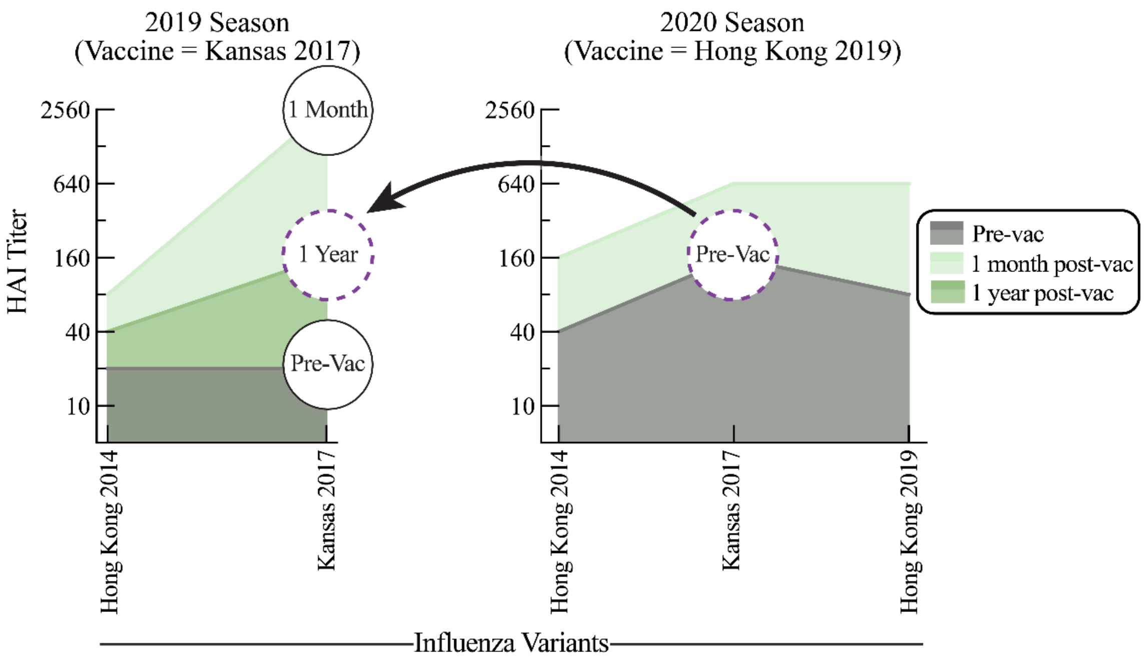

Figure S2. Pre-vac titers from the subsequent 2020 season determine the HAI titer 1 year post-vaccination from the 2019 season when prior vaccine strains are measured. Plots show hypothetical HAI titers from a single individual in a vaccine study carried out in both 2019 and 2020. Although the 2020 vaccine strain changed from H3N2 A/Kansas/14/2017 to H3N2 A/Hong Kong/45/2019, the pre-vaccination time point for H3N2 A/Kansas/14/2017 in 2020 provides the 1 year post-vaccination time point in the 2019 study. We assume the common scenario where each vaccine study measures current and prior vaccine strains, so that the 2019 study only measures H3N2 A/Hong Kong/4801/2014 and H3N2 A/Kansas/14/2017, whereas the 2020 study measures both prior vaccine strains and the current vaccine strain H3N2 A/Hong Kong/45/2019. This same method can be used across every season, and in seasons when the vaccine strain did not change (such as 2016-2017 and the 2017-2018 for H3N2), it can be used for studies that only measure the vaccine strain.

medRxiv preprint doi: https://doi.org/10.1101/2025.02.26.25322965; this version posted February 27, 2025. The copyright holder for this preprint
(which was not certified by peer review) is the author/funder, who has granted medRxiv a license to display the preprint in perpetuity.
It is made available under a CC-BY 4.0 International license .

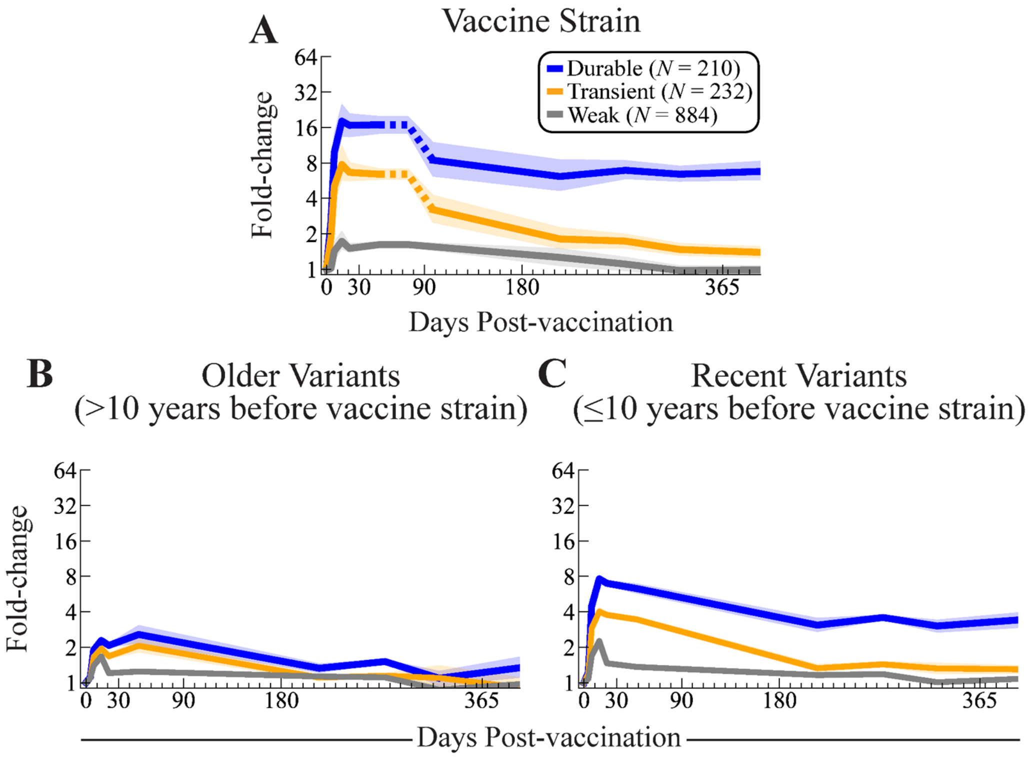

700 **Figure S3. Long-term dynamics of the vaccine response against variants.** Comparing the average vaccine response dynamics for durable, transient, and weak groups (A) against the vaccine, (B) variants circulating within 10 years of vaccination, and (C) variants circulating more than 10 years before vaccination.

medRxiv preprint doi: https://doi.org/10.1101/2025.02.26.25322965; this version posted February 27, 2025. The copyright holder for this preprint (which was not certified by peer review) is the author/funder, who has granted medRxiv a license to display the preprint in perpetuity.
It is made available under a CC-BY 4.0 International license .

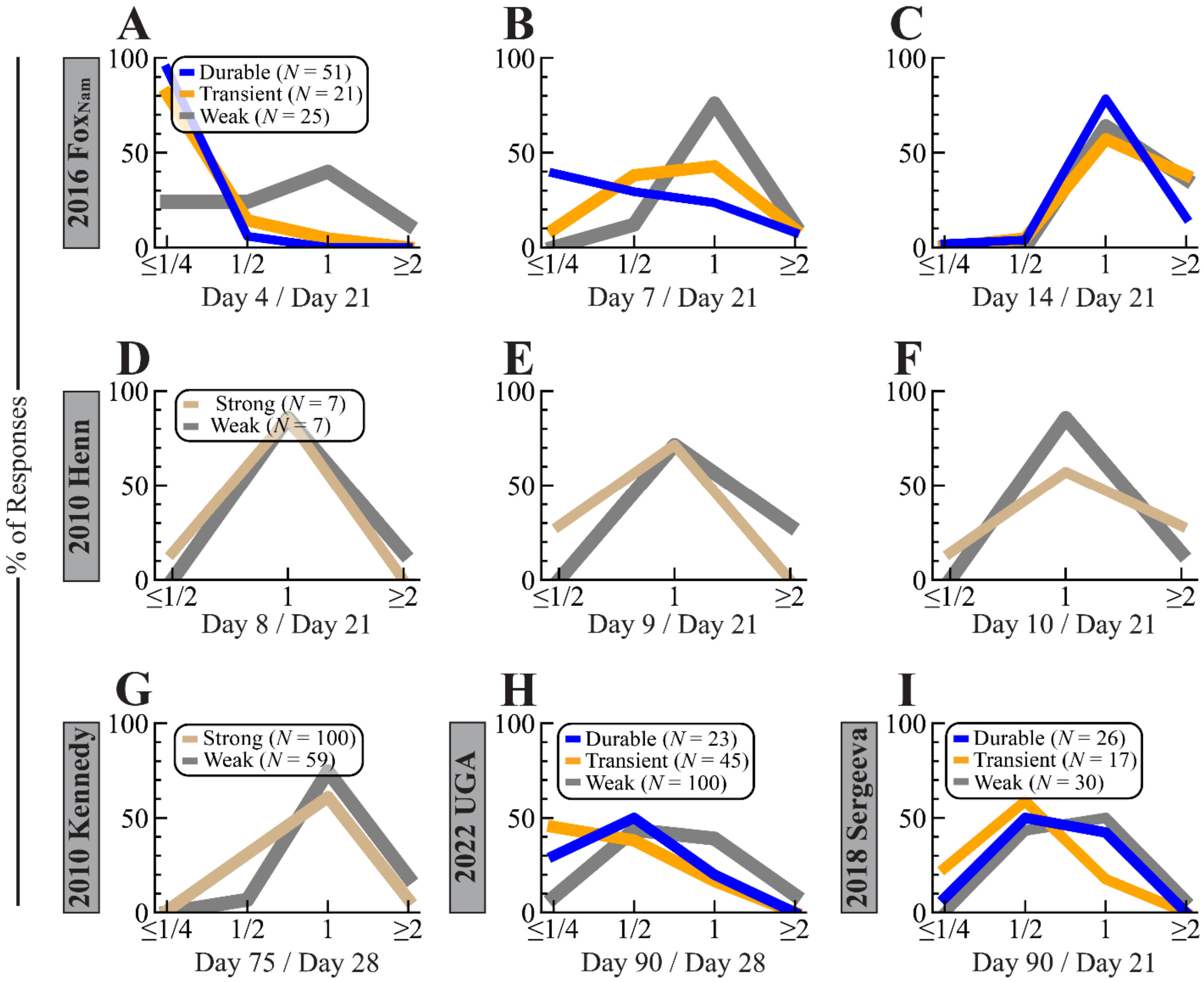

705

Figure S4. Comparing HAI titer at different time points in individual datasets used to determine peak timing. (A-C) Comparing HA titer against the vaccine strain (H3N2 A/Hong Kong/4801/2014) at early time points to day 21 within the 2016 Fox_{Nam} study.[9] (D-F) Comparing HAI titer against the vaccine strain (H3N2 A/Perth/16/2009) at early time points to day 21 within the 2010 Henn study.[14] (G) Comparing
710 HAI titer against the vaccine strain (H3N2 A/Perth/16/2009) at days 28 and 75 within the 2010 Kennedy study.[15] (H) Comparing HAI titer against the vaccine strain (H3N2 A/Darwin/9/2021) at days 28 and 90 within the 2022 UGA study.[13] (I) Comparing HAI titer against the vaccine strain (H3N2 A/Singapore/INFIMH-160019/2016) at days 21 and 90 within the 2018 Sergeeva study.

715

medRxiv preprint doi: https://doi.org/10.1101/2025.02.26.25322965; this version posted February 27, 2025. The copyright holder for this preprint (which was not certified by peer review) is the author/funder, who has granted medRxiv a license to display the preprint in perpetuity.
It is made available under a CC-BY 4.0 International license .

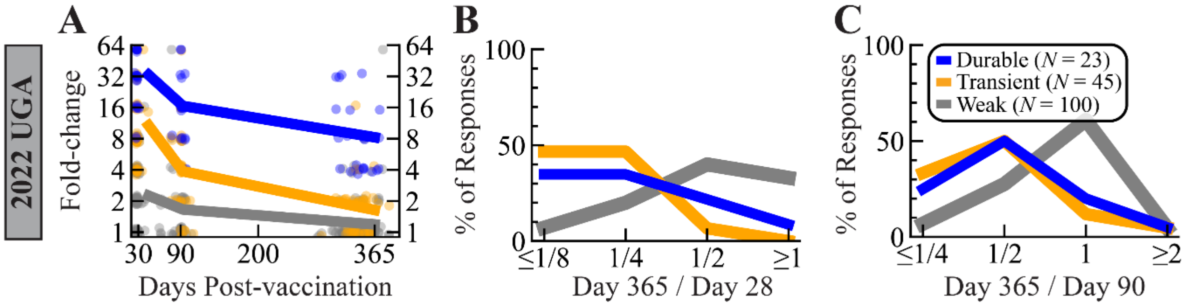

Figure S5. Long-term dynamics in the 2022 UGA study. (A) Fold-change against the vaccine strain (H3N2 A/Darwin/9/2021) at days 28, 90, and 365. HAI titer at (B) day 365 compared to day 28 or (C) day 365 compared to day 90. The number of individuals shown in the Panel C legend apply to all panels.

Table S1. Geometric mean HAI titers and fold-change across seasons at key time points. Statistics represent all subjects in each study. Fold-change is measured relative to pre-vac titers (and hence pre-vac fold-change=1 by definition).

Influenza Season (Studies from Table 1)	Pre-vac GMT (FC)	1 Month Post-vac GMT (FC)	1 Year Post-vac GMT (FC)
2014-15 (2014 $Hinojosa_V$)	183 (1.0x)	348 (1.9x)	304 (1.7x)
2016-17 (2016 Fox_{Nam}, 2016 Fox_{HCW}, 2016 UGA)	35 (1.0x)	169 (4.7x)	96 (2.7x)
2017-18 (2017 UGA)	100 (1.0x)	199 (2.0x)	74 (0.7x)
2018-19 (2018 UGA)	66 (1.0x)	123 (1.9x)	75 (1.1x)
2019-20 (2019 UGA)	11.7 (1.0x)	53 (4.5x)	20 (1.7x)
2020-21 (2020 UGA)	38 (1.0x)	87 (2.3x)	39 (1.0x)
2021-22 (2021 UGA)	32 (1.0x)	73 (2.3x)	52 (1.6x)
**Average**	**35 (1.0x)**	**102 (2.9x)**	**51 (1.5x)**

medRxiv preprint doi: https://doi.org/10.1101/2025.02.26.25322965; this version posted February 27, 2025. The copyright holder for this preprint (which was not certified by peer review) is the author/funder, who has granted medRxiv a license to display the preprint in perpetuity.
It is made available under a CC-BY 4.0 International license .

730 **Table S2. Studies without variants included in this work.** This table lists studies that were included in this work despite not having variants. 2010 Henn and 2010 Kennedy provided unique time points that were absent from **Table 1** and were therefore included. 2018 Sergeeva also lacked variants but was used as an existing study to validate our predictive analysis.

Year + Name of Study*	# Data Points	# Sera	# H3N2 Variants	Times Measured	H3N2 Vaccine Strain (Formulation)
2010 Henn[14]	168	14	1	[0, 1, 2, 3, 4, 5, 6,7, 8, 9, 10, 21]	A/Perth/16/2009 (FluLaval)
2010 Kennedy[15]	636	159	1	[0, 3, 28, 75]	A/Perth/16/2009 (Fluarix)
2018 Sergeeva[16]	436	73	1	[0,7,21, 90,180, 365]	A/Singapore/INFIMH-160019/2016 (Ultrix, Grippol Plus, or Sovigripp)

medRxiv preprint doi: https://doi.org/10.1101/2025.02.26.25322965; this version posted February 27, 2025. The copyright holder for this preprint (which was not certified by peer review) is the author/funder, who has granted medRxiv a license to display the preprint in perpetuity.
It is made available under a CC-BY 4.0 International license .

Table S3. Key statistics for the influenza H3N2 vaccine response. Separate statistics (and the number *N* of sera) are shown for the (A) pre-vaccination, (B) response initiation, (C) 1 month post-vaccination, (D) peak timing, and (E) 1 year post-vaccination responses. When datasets only measured responses out to 1 month post-vac, combined statistics are shown for transient and durable responders (collectively called strong responders). HAI fold-change (FC) is measured relative to pre-vaccination titers. As in Table 1, white backgrounds denote prior studies while gray backgrounds represent new studies introduced in this work.

(A)

Magnitude of the Pre-vaccination Response			
**Year + Name of Study**	**Weak GMT**	**Transient GMT**	**Durable GMT**
2014 Hinojosa$_V$	210 [*N*=36]	320 [*N*=1]	48 [*N*=4]
2016 Fox$_{Nam}$	47 [*N*=25]	40 [*N*=21]	28 [*N*=51]
2016 Fox$_{HCW}$	49 [*N*=31]	15 [*N*=10]	24 [*N*=8]
2016 UGA	53 [*N*=47]	28 [*N*=20]	28 [*N*=35]
2017 UGA	125 [*N*=118]	48 [*N*=20]	38 [*N*=12]
2018 UGA	82 [*N*=131]	25 [*N*=20]	33 [*N*=11]
2019 UGA	14 [*N*=182]	9 [*N*=80]	10 [*N*=58]
2020 UGA	48 [*N*=196]	19 [*N*=38]	12 [*N*=16]
2021 UGA	40 [*N*=118]	17 [*N*=22]	13 [*N*=15]
**Average**	**48**	**18**	**19**
2010 Kennedy	60 [*N*=59]	22 [*N*=100] (strong)	
2010 Henn	119 [*N*=7]	27 [*N*=7] (strong)	
2018 Sergeeva	38 [*N*=30]	18 [*N*=17]	9 [*N*=26]
2022 UGA	10 [*N*=100]	9 [*N*=45]	10 [*N*=23]
2018 Kennedy	43 [*N*=142]	26 [*N*=69] (strong)	

medRxiv preprint doi: https://doi.org/10.1101/2025.02.26.25322965; this version posted February 27, 2025. The copyright holder for this preprint (which was not certified by peer review) is the author/funder, who has granted medRxiv a license to display the preprint in perpetuity.
It is made available under a CC-BY 4.0 International license .

(B)

**Days post-vaccination when HAI increases**	
**Year + Name of Study**	**Days when strong responses reach ≥4x FC**
2016 Fox_{Nam}	Day 5-7<br>[*N*=72]
2010 Henn	Day 6-8<br>[*N*=7]
**Intersection**	**Day 6-7**

medRxiv preprint doi: https://doi.org/10.1101/2025.02.26.25322965; this version posted February 27, 2025. The copyright holder for this preprint (which was not certified by peer review) is the author/funder, who has granted medRxiv a license to display the preprint in perpetuity.
It is made available under a CC-BY 4.0 International license .

(C)

**Magnitude of 1 Month (Peak) Response**			
**Year + Name of Study**	**Weak GMT (FC)**	**Transient GMT (FC)**	**Durable GMT (FC)**
2014 Hinojosa$_V$	291 (1.4x) [*N*=36]	1280 (4.0x) [*N*=1]	1280 (26.9x) [*N*=4]
2016 Fox$_{Nam}$	66 (1.4x) [*N*=25]	280 (7.0x) [*N*=21]	559 (19.6x) [*N*=51]
2016 Fox$_{HCW}$	73 (1.5x) [*N*=31]	113 (7.5x) [*N*=10]	226 (9.5x) [*N*=8]
2016 UGA	71 (1.3x) [*N*=47]	149 (5.3x) [*N*=20]	326 (11.7x) [*N*=35]
2017 UGA	167 (1.3x) [*N*=118]	269 (5.7x) [*N*=20]	678 (18.0x) [*N*=12]
2018 UGA	112 (1.4x) [*N*=131]	126 (5.1x) [*N*=20]	363 (11.0x) [*N*=11]
2019 UGA	31 (2.2x) [*N*=182]	69 (8.0x) [*N*=80]	185 (19.1x) [*N*=58]
2020 UGA	79 (1.6x) [*N*=196]	105 (5.6x) [*N*=38]	167 (14.1x) [*N*=16]
2021 UGA	62 (1.5x) [*N*=118]	83 (5.0x) [*N*=22]	232 (17.6x) [*N*=15]
**Average**	**77 (1.6x)**	**112 (6.4x)**	**313 (16.3x)**
2010 Kennedy	69 (1.2x) [*N*=59]	186 (8.6x) [*N*=100] (strong)	
2010 Henn	145 (1.2x) [*N*=7]	390 (14.5x) [*N*=7] (strong)	
2018 Sergeeva	52 (1.4x) [*N*=30]	136 (7.7x) [*N*=17]	220 (23.9x) [*N*=26]
2022 UGA	24 (2.3x) [*N*=100]	95 (10.7x) [*N*=45]	340 (33.0x) [*N*=23]
2018 Kennedy	66 (1.5x) [*N*=142]	134 (5.2x) [*N*=69] (strong)	

medRxiv preprint doi: https://doi.org/10.1101/2025.02.26.25322965; this version posted February 27, 2025. The copyright holder for this preprint (which was not certified by peer review) is the author/funder, who has granted medRxiv a license to display the preprint in perpetuity.
It is made available under a CC-BY 4.0 International license .

(D)

Days post-vaccination when the peak response occurs		
**Year + Name of Study**	**Days for Peak Start**	**Days for Peak End**
2016 Fox$_{Nam}$	Days 7-14 [*N*=72]	-
2010 Henn	Days 10-21 [*N*=7]	-
2010 Kennedy	-	Days >75 [*N*=100]
2022 UGA	-	Days <90 [*N*=68]
**Intersection**	**Days 10-14**	**Days 76-90**

medRxiv preprint doi: https://doi.org/10.1101/2025.02.26.25322965; this version posted February 27, 2025. The copyright holder for this preprint (which was not certified by peer review) is the author/funder, who has granted medRxiv a license to display the preprint in perpetuity.
It is made available under a CC-BY 4.0 International license .

750 **(E)**

Magnitude of 1 Year Response			
**Year + Name of Study**	**Weak GMT (FC)**	**Transient GMT (FC)**	**Durable GMT (FC)**
2014 Hinojosa$_V$	274 (1.3x) [*N*=36]	320 (1.0x) [*N*=1]	761 (16.0x) [*N*=4]
2016 Fox$_{Nam}$	59 (1.2x) [*N*=25]	75 (1.9x) [*N*=21]	210 (7.4x) [*N*=51]
2016 Fox$_{HCW}$	60 (1.2x) [*N*=31]	28 (1.9x) [*N*=10]	123 (5.2x) [*N*=8]
2016 UGA	86 (1.6x) [*N*=47]	53 (1.9x) [*N*=20]	173 (6.2x) [*N*=35]
2017 UGA	68 (0.5x) [*N*=118]	57 (1.2x) [*N*=20]	240 (6.3x) [*N*=12]
2018 UGA	78 (1.0x) [*N*=131]	29 (1.2x) [*N*=20]	265 (8.0x) [*N*=11]
2019 UGA	18 (1.2x) [*N*=182]	13 (1.5x) [*N*=80]	63 (6.5x) [*N*=58]
2020 UGA	41 (0.9x) [*N*=196]	24 (1.3x) [*N*=38]	70 (5.9x) [*N*=16]
2021 UGA	54 (1.3x) [*N*=118]	26 (1.6x) [*N*=22]	106 (8.0x) [*N*=15]
**Average**	**48 (1.0x)**	**26 (1.5x)**	**131 (6.8x)**
2018 Sergeeva	29 (0.8x) [*N*=30]	25 (1.4x) [*N*=17]	96 (10.4x) [*N*=26]
2022 UGA	12 (1.2x) [*N*=100]	14 (1.6x) [*N*=45]	85 (8.2x) [*N*=23]
